# Mitochondrial Nicotinic Acetylcholine Receptors Support Liver Cells Viability After Partial Hepatectomy

**DOI:** 10.3389/fphar.2018.00626

**Published:** 2018-06-13

**Authors:** Kateryna Uspenska, Olena Lykhmus, Maria Obolenskaya, Stephanie Pons, Uwe Maskos, Serhiy Komisarenko, Maryna Skok

**Affiliations:** ^1^Laboratory of Cell Receptors Immunology, O. V. Palladin Institute of Biochemistry, Kiev, Ukraine; ^2^System Biology Group, Institute of Molecular Biology and Genetics, Kiev, Ukraine; ^3^Integrative Neurobiology of Cholinergic Systems, Institut Pasteur, Paris, France

**Keywords:** nicotinic acetylcholine receptor, mitochondria, knockout mice, partial hepatectomy, cytochrome *c*, mitochondrial kinases

## Abstract

Nicotinic acetylcholine receptors (nAChRs) expressed on the cell plasma membrane are ligand-gated ion channels mediating fast synaptic transmission, regulating neurotransmitter and cytokine release and supporting the viability of many cell types. The nAChRs expressed in mitochondria regulate the release of pro-apoptotic factors, like cytochrome *c*, in ion channel-independent manner. Here we show that α3β2, α7β2, and α9α10 nAChR subtypes are up-regulated in rat liver mitochondria 3–6 h after partial hepatectomy resulting in increased sustainability of mitochondria to apoptogenic effects of Ca^2+^ and H_2_O_2_. In contrast, laparotomy resulted in down-regulation of all nAChR subunits, except α9, and decreased mitochondria sustainability to apoptogenic effects of Ca^2+^ and H_2_O_2_. Experiments performed in liver mitochondria from α3+/-, α7-/-, β4-/-, α7β2-/-, or wild-type C57Bl/6J mice demonstrated that the decrease of α3 or absence of α7 or α7/β2 subunits in mitochondria is compensated with β4 and α9 subunits, which could be found in α3β4, α4β4, α9β4, and α9α10 combinations. Mitochondria from knockout mice maintained their sustainability to Ca^2+^ but were differently regulated by nAChR subtype-specific ligands: PNU-282987, methyllycaconitine, dihydro-β-erythroidine, α-conotoxin MII, and α-conotoxin PeIA. It is concluded that mitochondrial nAChRs play an important role in supporting the viability of hepatic cells and, therefore, may be a pharmacological target for pro-survival therapy. The concerted action of multiple nAChR subtypes controlling either CaKMII- or Src-dependent signaling pathways in mitochondria ensures a reliable protection against apoptogenic factors of different nature.

## Introduction

Nicotinic acetylcholine receptors are ligand-gated ion channels initially discovered in neuro-muscular synapses and further found in neurons and in many non-excitable cells (Skok, 2002; Kalamida et al., 2007; Kawashima and Fujii, 2008; Gotti et al., 2009). The classical idea on the nAChR involvement in fast synaptic transmission has evolved along with the understanding of universal and evolutionary ancient kind of acetylcholine signaling in the living nature (Wessler and Kirkpatrick, 2008). Now it is clear that acetylcholine and its receptors (both nicotinic and muscarinic) appeared in evolution long before the development of nerve and muscle systems and were initially intended to regulate the vital functions of primitive cells like survival, proliferation and adhesion (Kawashima and Fujii, 2008). The prototypes of nAChRs were found in bacteria where they respond to the changes in the environmental pH (Bocquet et al., 2007).

Structurally, the nAChRs are homo- or hetero-pentamers composed of established combinations of ten α (α1 to α10) and four β (β1 to β4) subunits; muscular nAChRs contain 2α1, β1, γ and δ or 𝜀 subunits (Kalamida et al., 2007). Binding of agonist results in concerted conformational movement of the subunits within the pentamer leading to ion channel opening (gating) (Changeux, 2012). However, certain kinds of nAChR signaling, especially in non-excitable cells, seem not to require gating as such and are, therefore, ion-independent (Skok, 2009). Probably, this is the most ancient type of cholinergic signaling not related to significant changes in the cell membrane polarity.

The important step in the understanding of the universal character of cholinergic signaling was the discovery of functional nAChRs in mitochondria (Gergalova et al., 2012). Previously, we reported that several nAChR subtypes are found in the outer membrane of mouse mitochondria in a tissue-specific manner and in close relation to voltage-dependent anion channels (VDACs) (Gergalova et al., 2012; Lykhmus et al., 2014). The function of mitochondrial nAChRs appeared to be ion channel-independent, because they engage intramitochondrial kinases under the effect of agonists, antagonists and even positive allosteric modulators (Gergalova et al., 2014; Uspenska et al., 2017). The main function of mitochondrial nAChRs known by now is to regulate the early stages of mitochondria-driven apoptosis like cyt *c* release (reviewed in Skok et al., 2016). In contrast, nicotine effects on mitochondria respiratory chain appeared to be receptor-independent (Cormier et al., 2001). The nAChRs found in the brain mitochondria are modulated by inflammation (Lykhmus et al., 2016) and influence accumulation of amyloid peptides (1–40)/(1–42) in an experimental model of Alzheimer-like pathology in mice (Lykhmus et al., 2015). Others have shown that malignant cell transformation is accompanied by the increase of mitochondrial nAChR number (Grando, 2014), that was related to increased viability of cancer cells. However, the real value of cholinergic signaling in mitochondria is still far from being completely understood.

In the present study, we used two experimental models: rats subjected to PHE and knockout mice lacking certain nAChR subunits. The first model demonstrated the role of mitochondrial nAChRs in supporting the cell viability during the priming phase of liver regeneration. The second model allowed us to reveal compensatory mechanisms occurring in the absence of certain mitochondrial nAChR subtypes.

## Materials and Methods

### Materials

All reagents were of chemical grade and were purchased from Sigma-Aldrich (Saint Louis, United States), unless specially indicated. Antibodies against α7(1–208) (Lykhmus et al., 2010), α3(181–192), α4(181–192), α7(179–190) (Skok et al., 1999), α9(11–23) (Koval et al., 2011), β2(190–200) or β4(190–200) (Koval et al., 2004) nAChR fragments were obtained and characterized previously in our lab. Antibody against α10(404–417) (Lips et al., 2002) was a kind gift of Dr. W. Kummer (Justus Liebig University Giessen, Giessen, Germany). The antibodies were biotinylated according to standard procedure (Harlow and Lane, 1988). Rabbit cyt *c*-specific antibodies were generated as previously described (Gergalova et al., 2014). Conotoxin MII, synthesized according to Surin et al. (2012) and α-conotoxin PeIA, synthesized as described (Koval et al., 2011) was a kind gift of Prof. V. Tsetlin (Shemyakin-Ovchinnikov Institute of Bioorganic Chemistry, RAS, Russia). Dexalgin (analog of *Dexketoprofen*, (2*S*)-2-[3-(benzoyl)phenyl]propanoic acid) was purchased from the public drugstore in Kiev.

### Animals and Procedures

Animals were kept in the animal facilities of Institut Pasteur, Paris, France and O. V. Palladin Institute of Biochemistry, Kiev, Ukraine. They were housed in quiet, temperature-controlled rooms and provided with water and food pellets *ad libitum*. All procedures conformed with the guidelines of the Center National de la Recherche Scientifique or Palladin Institute’s IACUC. Before starting the experiments, the protocols were approved by the IACUC.

Age-matched C57BL/6J wild-type (WT) and knockout mice lacking either α7 (Orr-Urtreger et al., 1997), α7 and β2 or β4 (Xu et al., 1999b) nAChR subunits, 20–22 g, of both genders were used. Since homozygous α3-/- mice do not survive (Xu et al., 1999a), heterozygous α3+/- mice were used instead. Before removing the liver mice were sacrificed by cervical dislocation.

Male Wistar rats (200–250 g) were operated under ether anesthesia according to standard procedures (Higgins and Anderson, 1931; Tannuri et al., 2007). The animal’s abdomen was opened carefully. In PHE, medial and left lateral liver lobes were resected and further used as controls compared to regenerating liver. Then the abdomen was closed with a single-layer running suture. In LT, no liver parts were removed and the abdomen was closed similarly to PHE. Following surgery, the operated animals obtained a single intramuscular injection of dexalgin (0.33 mg/kg), they were returned to their cages and fed regular diets and water *ad libitum*. The animals were sacrificed by decapitation under ether anesthesia 3, 6, 12, or 24 hours after the operation.

### Mitochondria Purification

Mitochondria were isolated from rodent liver by differential ultracentrifugation according to standard published procedures (Sottocasa et al., 1967; Gergalova et al., 2012). In brief, the liver was homogenized in the separation medium containing 10 mM HEPES, 1 mM EGTA, and 250 mM sucrose, pH 7.4, 4°C. The primary homogenate was centrifuged at 1,500 × *g* at 4°C for 10 min, the pellet being considered as non-mitochondrial fraction. The supernatant containing mitochondria was centrifuged at 8,000 × *g* at 4°C for 10 min and the pellet was washed by additional centrifugation at similar conditions. The purity of mitochondria and mitochondria-depleted fractions was assessed by ELISA using the antibodies against nuclear-specific lamin B1, mitochondria-specific VDAC or endoplasmic reticulum-specific IRE-1α as described (Uspenska et al., 2017). The purified live mitochondria were used for functional cyt *c* release studies. To prepare detergent lysates, the pellets of both fractions were frozen at -20°C, thawed and treated with lysing buffer (0.01 M Tris–HCl, pH 8.0; 0.14 NaCl; 0.025% NaN_3_; 1% Tween-20 and protease inhibitors cocktail) for 2 h on ice upon intensive stirring. The resulting lysates were pelleted by centrifugation (20 min at 20,000 × *g*). The protein concentration was established by using the BCA Protein Assay kit (Thermo Fisher Scientific, Rockford, United States).

### Cytochrome *c* Release Studies

The purified live mitochondria were incubated with different concentrations of CaCl_2_ or 0.5 mM H_2_O_2_ in the presence or absence of various nAChR ligands at room temperature (RT), for 5 min, and immediately pelleted by centrifugation (10 min, 7,000 × g at 4°C). The incubation medium contained 10 mM HEPES, 125 mM KCl, 25mM NaCl, 5 mM sodium succinate, and 0.1 mM Pi(K), pH 7.4. The nAChR ligands: PNU-282987 (30 nM), MLA (50 nM), DhβE (1 μM), α-conotoxin MII (1 nM), and α-conotoxin PeIA (2.5, 5, and 25 nM) were added to the incubation medium 2 min prior to the apoptogenic agents. The mitochondria supernatants were collected and tested for the presence of cyt c by sandwich assay as described (Gergalova et al., 2012, 2014). Experimental values of OD_490__nm_ were within the linear part of the calibration curve built with bovine cyt *c*.

### Sandwich Assays

To determine the type of nAChR subunits in mitochondria and mitochondria-depleted fractions, 96-well plates (Nunc Maxisorb, Roskilde, Denmark) were coated with α7(1–208)-specific antibody (30 μg/ml at 4°C, overnight), subsequently blocked with 1% BSA/PBS, and the detergent lysates of the corresponding fractions (100 μg/ml) were applied into coated wells for 2 h at 37°C. Then, the plates were rinsed with water and the bound antigen was revealed with biotinylated α3(181–192)-, α4(181–192)-, α7(179–190)-, α9(11–23)-, α10(404–417)-, β2(190–200), or β4(190–200)-specific antibodies applied for additional 2 h, followed by NeutrAvidin-peroxidase conjugate and *o*-phenylendiamine-containing substrate solution. The Stat-Fax2100 Microplate reader (Awareness Technology, Inc., FL, United States) was used to determine the optical density of the samples at 490 nm absorbance (OD490 nm). All antibodies have been preliminary titrated on corresponding antigenic peptides and the doses (dilutions) have been selected according to titration curves: 1:200 for α3-specific, 1:80 for α4-specific, 1:300 for α7-specific, 1:250 for α9-specific, 1:250 for α10-specific, 1:500 for β2-specific, and 1:200 for β4-specific, assuming that the initial concentration of all antibodies was 2 mg/ml.

To determine the nAChR subunit combinations, the plates were coated with α3-, α4-, α9-, α10- or β4-specific antibody (30 μg/ml) and the bound nAChRs from mitochondria detergent lysate were revealed with biotinylated α9(11–23)- or β4(190–200)-specific antibodies similarly to procedure described above.

### Statistical Analysis

We used five mice per genotype in experiments with knockout mice and three rats per time point in PHE and LT experiments. All ELISAs were performed in triplicates and the average values for each mouse or rat were used to calculate means and SE. Statistical analysis was performed using Student’s *t*-test; the difference was considered significant at *p* < 0.05.

## Results

### Mitochondrial nAChRs Are Involved in Liver Regeneration After PHE

**FIGURE 1:**
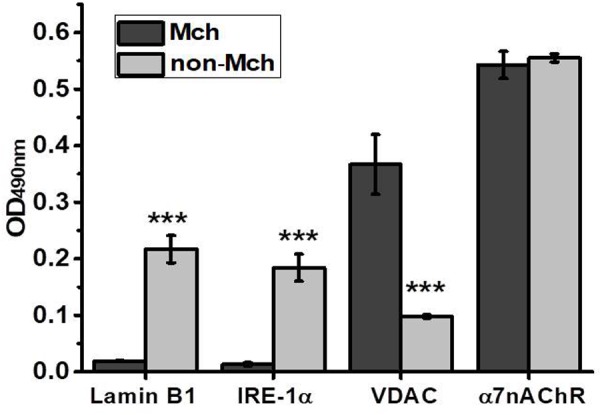
ELISA assay characterizing the purity of mitochondria (Mch) vs. mitochondria-depleted (non-Mch) rat liver fractions. Each column corresponds to mean ± SE, *n* = 3; ^∗∗∗^*p* < 0.0005 compared to Mch fraction.

Partial hepatectomy is a unique experimental model enabling to investigate the early events of tissue regeneration. It provides relevant and individual controls, the parts of liver resected in the course of surgery, to be compared with the remaining parts undergoing regeneration for various periods of time. Additional control is provided by the control surgery, LT, which allows excluding the effects of skin damage, anesthesia and inflammation also present during hepatectomy. In our experiments, to minimize the number of animals used, we at first performed a time-dependent study using one animal per time point and then added more rats for the most important time points (3 and 6 h for PHE and 6 and 12 h for LT) to obtain statistically significant results. Mitochondria purified from the liver of rats were immediately examined in the functional assay of cyt *c* release under the effect of 0.9 μM Ca^2+^ or 0.5 mM H_2_O_2_ and were further studied in Sandwich ELISA for the presence of various nAChR subunits. A similar ELISA assay was performed in the mitochondria-depleted liver fraction. The purity of mitochondrial and mitochondria-depleted preparations was characterized by ELISA using the antibodies against various intracellular compartments/organelles (Uspenska et al., 2017). As shown in **Figure 1**, the mitochondrial fraction was positive for mitochondria-specific marker VDAC (Colombini, 2004) and was negative for the nuclear-specific marker α-lamin B1 (Gruenbaum et al., 2000) and ER-specific marker IRE-1α (Chen and Brandizzi, 2013), while the mitochondria-depleted fraction, *vice versa*, was positive for lamin B1 and IRE-1α, but negative for VDAC. In contrast, the α7 nAChR content was similar in both fractions indicating its equal presence in either mitochondria or non-mitochondrial cell compartments. We did not use the markers of the plasma membrane assuming that plasma membrane nAChRs comprise only a small part (about 15%) of the whole intracellular pool (Sallette et al., 2005).

**FIGURE 2:**
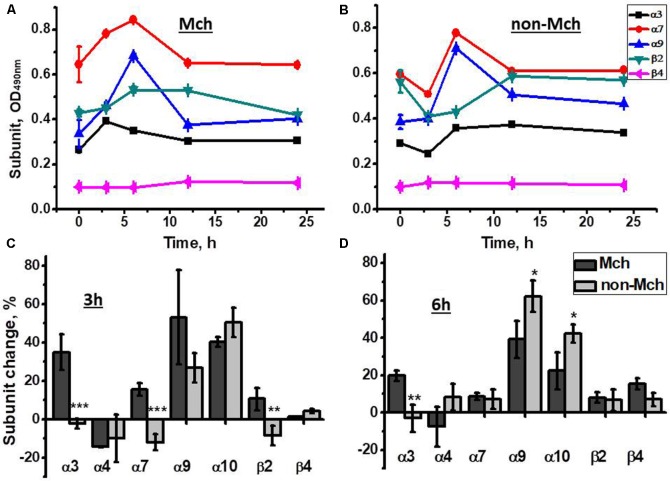
The changes in nAChR subunits content in the mitochondria (Mch) and mitochondria-depleted (non-Mch) liver fractions of rats subjected to PHE. **(A,B)** Absolute OD values, one rat per each time point; **(C,D)** normalized values for three rats per each time point compared to the section of the liver removed during hepatectomy (mean ± SE). ^∗^*p* < 0.05; ^∗∗^*p* < 0.005; ^∗∗∗^*p* < 0.0005 compared to mitochondrial fraction.

As shown in **Figure 2A**, an increase of α3, α7, and α9 nAChR subunits was observed in liver mitochondria already 3 h after hepatectomy reaching a maximum at 6 h. In contrast, in the liver fraction depleted of mitochondria, the decrease of corresponding subunits was found 3 h after surgery, while the peak increase was observed at the 6 h time point (**Figure 2B**). Later (12 and 24 h) the level of both mitochondrial and non-mitochondrial nAChRs was decreased reaching almost pre-operational state values. These data were confirmed with a statistically relevant number of animals for the 3 and 6 h time points and using a wider range of nAChR subunit-specific antibodies. As shown in **Figures 2C,D**, mitochondria up-regulated α3, α7, α9, α10, and β2 subunits at 3 h, whereas α7 and β2 subunits were decreased in the non-mitochondrial fraction at this time point. The α9 and α10 subunits were increased in both liver fractions. At 6 h, the increase of α4, α7, α9, β2, and β4 subunits was observed in the non-mitochondria fraction and some β4 subunits appeared in mitochondria. These data suggested a redistribution of the liver nAChR pool in favor of mitochondria 3 h after hepatectomy followed by *de novo* nAChR synthesis at 6 h. Taking into account the established subunit combinations, these data indicate that the early stage of liver regeneration was accompanied by up-regulation of α3β2, α7β2, and α9α10 nAChR subtypes in mitochondria.

**FIGURE 3:**
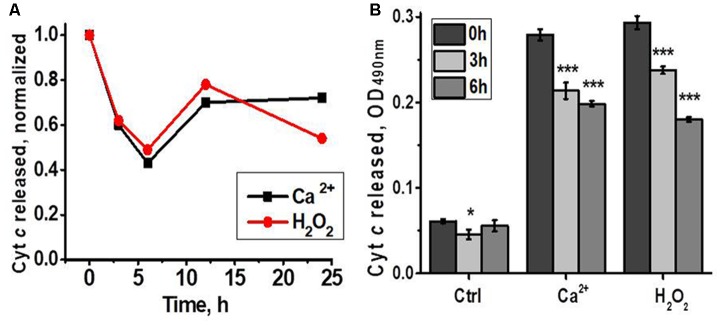
Cyt *c* released from liver mitochondria of hepatectomized rats under the effect of either Ca^2+^ (0.9 μM) or H_2_O_2_ (0.5 mM). **(A)** Normalized data for one rat per each time point; **(B)** raw data for three rats per each time point; mean ± SE; ^∗^*p* < 0.05; ^∗∗∗^*p* < 0.0005 compared to pre-operation state (0 h).

According to the data of functional assay, mitochondria of regenerating liver released less cyt *c* in response to Ca^2+^ or H_2_O_2_ 3 and 6 h after hepatectomy, while later there was no difference with the section of the liver removed during the hepatectomy (**Figure 3**). Therefore, up-regulated nAChR subtypes underlied mitochondria increased sustainability to apoptogenic stimuli.

**FIGURE 4:**
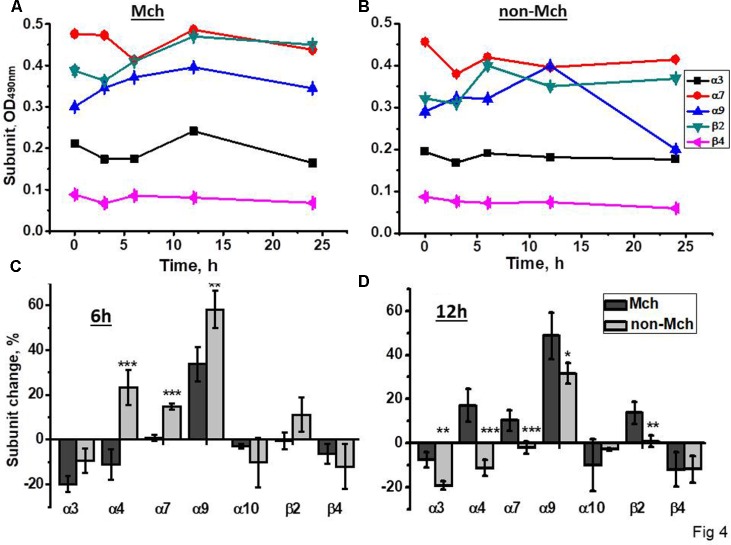
The changes in nAChR subunits content in the mitochondria (Mch) and mitochondria-depleted (non-Mch) liver fractions of rats subjected to LT. **(A,B)** Absolute OD values, one rat per each time point; **(C,D)** normalized values for three rats per each time point compared to the section of the liver removed during hepatectomy (mean ± SE). ^∗^*p* < 0.05; ^∗∗^*p* < 0.005; ^∗∗∗^*p* < 0.0005 compared to mitochondrial fraction.

A similar experimental schedule was applied for rats subjected to control surgery –LT. As shown in **Figure 4**, all nAChR subunits, except α9, were decreased in mitochondria 3 and 6 h after surgery, the restoration of α4, α7, and β2 was observed at 12 h only. In the mitochondria-depleted liver fraction, α4, α7, and β2 subunits were increased at 6 h already and, again, the main increase was found for α9 subunits, but not for α10 subunits. Therefore, LT was accompanied by the down-regulation of all liver nAChRs except α9-containing ones, which increased; the restoration of α4β2 and α7β2 nAChR subtypes started in the non-mitochondrial fraction 6 h after surgery.

**FIGURE 5:**
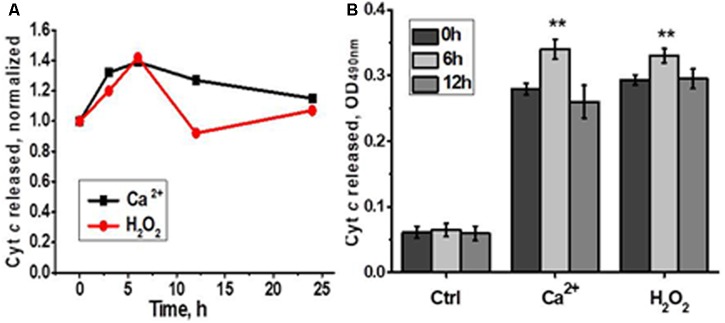
Cyt *c* released from liver mitochondria of laparotomized rats under the effect of either Ca^2+^ (0.9 μM) or H_2_O_2_ (0.5 mM). **(A)** Normalized data for one rat per each time point; **(B)** raw data for three rats per each time point; mean ± SE; ^∗∗^*p* < 0.005 compared to pre-operation state (0 h).

In the functional assay, liver mitochondria of laparotomised rats released more cyt *c* in response to either Ca^2+^ or H_2_O_2_ 6 h after surgery compared to mitochondria of non-operated rats (**Figure 5**). These data indicated that LT initially made liver mitochondria more sensitive to apoptogenic influence and up-regulated α9 nAChRs were insufficient to fully compensate the decrease of other nAChR subtypes.

Therefore, the effect of LT was different from that of PHE indicating that different physiological processes were stimulated by these two types of intervention. The only common feature was a significant increase of α9 nAChR subunits in both mitochondria and mitochondria-depleted fractions observed shortly after surgery. However, after PHE, the α9 up-regulation was accompanied by the increase of α10 subunits indicating the appearance of heteromeric α9α10 nAChRs, while after LT α9 subunits, most probably, were included in homomeric receptors.

**FIGURE 6:**
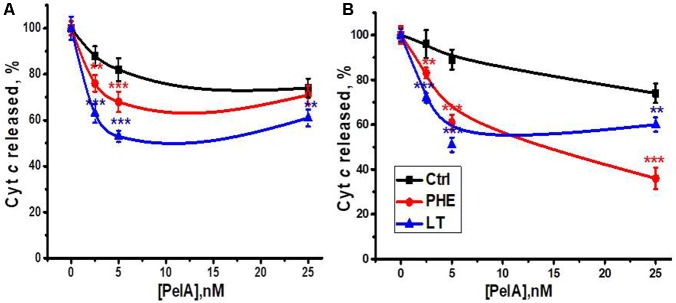
The effect of α-conotoxin PeIA on Ca^2+^ (0.9 μM)-stimulated **(A)** or H_2_O_2_ (0.5 mM)-stimulated **(B)** cyt *c* release from mitochondria of non-operated (Ctrl), hepatectomized (PHE) or laparotomized (LT) rats 6 h after surgery. Each point corresponds to mean ± SE; *n* = 3; ^∗∗^*p* < 0.005; ^∗∗∗^*p* < 0.0005 compared to corresponding PeIA concentration for non-operated rats mitochondria.

The α-conotoxin PeIA is known to specifically target α9-containing nAChR subtypes (McIntosh et al., 2005). We compared the effect of α-conotoxin PeIA on mitochondria obtained from the section of the liver removed during the hepatectomy, regenerating liver and the liver after LT. As shown in **Figure 6**, α-conotoxin PeIA was poorly efficient in mitochondria of the liver removed during the hepatectomy and much more efficient in mitochondria of either PHE or LT liver, that was in accord with significant up-regulation of α9-containing nAChRs upon both types of surgery. In the Ca^2+^-stimulated cyt *c* release assay, α-conotoxin PeIA was more efficient in mitochondria from LT than PHE rats (50 vs. 30% of inhibition at 5nM, **Figure 6A**). However, when cyt *c* release was stimulated by H_2_O_2_, 25 nM conotoxin PeIA was more efficient in mitochondria from PHE than from LT rats (60 vs. 40% of inhibition, respectively, **Figure 6B**). Five nanomolar conotoxin PeIA was equally efficient against either Ca^2+^ or H_2_O_2_ in mitochondria of LT rats (about 50% of inhibition). Taking into account that (as shown above) PHE and LT resulted in up-regulation of different α9-containing nAChR subtypes in mitochondria, these data indicate that homomeric α9 receptors (LT) regulated both Ca^2+^- and H_2_O_2_-stimulated cyt *c* release from mitochondria, while activating α9α10 ones (PHE), with higher dose of α-conotoxin PeIA, attenuated preferentially H_2_O_2_-stimulated cyt *c* release.

### Mitochondria of α7-/-, α7β2-/-, and α3+/- Mice Up-Regulate α9 and β4 nAChR Subunits to Maintain Their Sustainability to Ca^2+^

The aim of this part of the study was to reveal if the absent nAChR subtypes are substituted with other ones in mitochondria of knockout animals and whether such substitution influences mitochondria sustainability to apoptogenic factors.

**FIGURE 7:**
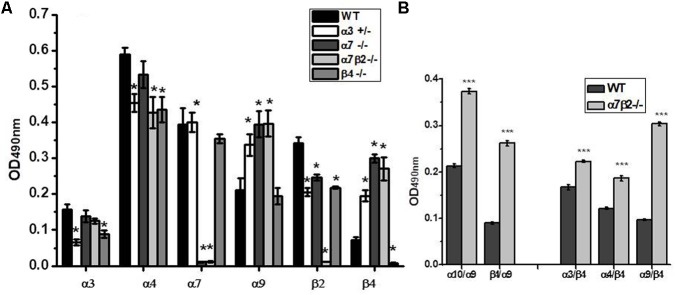
The nAChR subunit composition **(A)** and subunit combinations **(B)** in mitochondria of the wild-type (WT) and α7β2–/– mice. Each column corresponds to mean ± SE, five mice per genotype; ^∗^*p* < 0.05 compared to WT. The designations along abscissa correspond to the antibody combinations in sandwich ELISA: e.g., α9/β4 (α9-specific capture antibody and β4-specific detecting antibody) and β4/α9 (β4-specific capture antibody and α9-specific detecting antibody). ^∗∗∗^*p* < 0.0005 compared to WT.

Sandwich ELISA assay with nAChR subunit-specific antibodies confirmed the absence of corresponding nAChR subunits in mitochondria of knockout mice and the decrease of α3 subunits in the α3+/- heterozygote (**Figure 7A**). The α4 subunits were down-regulated in the absence of either β2 or β4 subunits, and β2 subunits were down-regulated in the absence of α7 or the decrease of α3 subunits, thus confirming the presence of heteromeric α3β2, α4β2, and α7β2 nAChRs in mouse liver mitochondria. Most interestingly, the lack of α7, α7β2 or the decrease of α3 subunits resulted in significant up-regulation of α9 and β4 nAChR subunits.

To reveal the nAChR subtypes formed upon up-regulation of α9 and β4 subunits in mitochondria of knockout mice, we performed sandwich ELISA where the nAChR molecule was captured by one subunit-specific antibody and was detected with the antibody against another nAChR subunit. This assay has been developed in our laboratory and was successfully used to show the presence of α7β2 nAChRs in mitochondria and their substitution with α7β4 in the absence of the β2 subunit (Lykhmus et al., 2014). As shown in **Figure 7B**, the absence of α7 and β2 subunits resulted in up-regulation of α9α10, α3β4, and α4β4 subunit combinations. To our surprise, we also observed a clear signal for the α9β4 combination (in both antibody orientations) and its significant increase in mitochondria of α7β2-/- mice. These data demonstrated an intriguing possibility of α9β4 combination in mitochondria.

**FIGURE 8:**
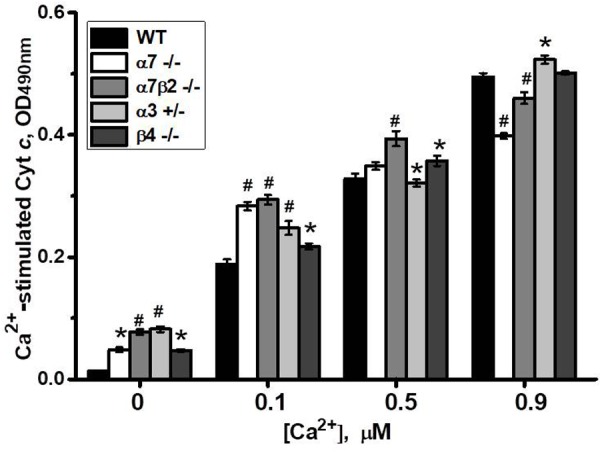
Cyt *c* release from mitochondria of the wild-type (WT) or knockout mice in response to different Ca^2+^ doses. Each column corresponds to mean ± SE, five mice per genotype; ^∗^*p* < 0.05; ^#^*p* < 0.0005 compared to WT.

To study if the absence of any nAChR subtype affects mitochondria sustainability to Ca^2+^ we tested liver mitochondria of knockout mice in a cyt *c* release assay with increasing Ca^2+^ doses. As shown in **Figure 8**, mitochondria of all knockout mice released small amounts of cyt *c* even without Ca^2+^ addition. With low Ca^2+^ dose (0.1 μM), mitochondria of α7-/-, α7β2-/- and, to a lesser extent, α3+/- mice released more cyt *c* compared to mitochondria of WT mice. With 0.5 μM Ca^2+^, significant difference remained only for α7β2-/- mitochondria, and with 0.9 μM Ca^2+^, α7-/- and α7β2-/- mitochondria were even more resistant to Ca^2+^ than the WT ones. These data suggested that the nAChR subtypes expressed in mitochondria of knockout mice were less efficient against low, but more efficient against high Ca^2+^ doses compared to the nAChRs of the WT mitochondria.

**FIGURE 9:**
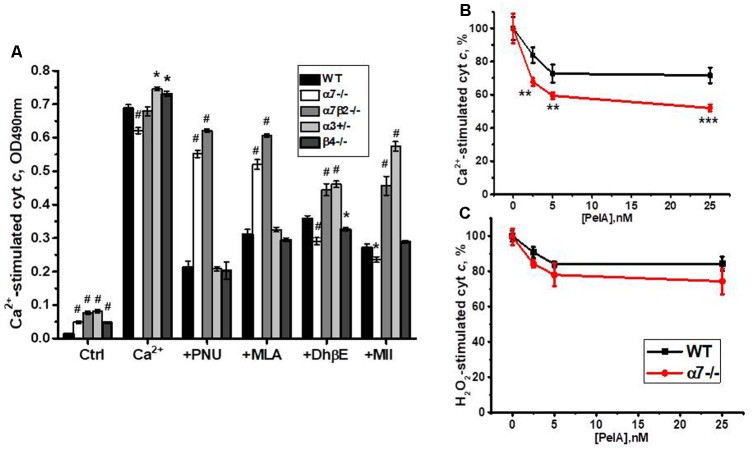
The effect of PNU 282987 (1 μM), MLA (50 nM), DhβE (1 μM), and α-conotoxin MII (1 nM) **(A)** or α-conotoxin PeIA **(B,C)** on Ca^2+^ (0.9 μM)-stimulated **(A,B)** and H_2_O_2_ (0.5 mM)-stimulated **(C)** cyt *c* release from mitochondria of the wild-type (WT) and knockout mice. Each column **(A)** or point **(B,C)** corresponds to mean ± SE, three to five mice per genotype; ^∗^*p* < 0.05; ^∗∗^*p* < 0.005; ^∗∗∗^ or ^#^*p* < 0.0005 compared to WT.

Previously we reported that cyt *c* release from mitochondria can be inhibited by either α7-specific agonist PNU282987 or, to a lesser extent, antagonist methyllicaconitine (MLA), as well as by α4β2-specific DhβE or α3β2-specific α-conotoxin MII (Gergalova et al., 2014). When tested with nAChR subtype-specific ligands, cyt *c* release from mitochondria of α7-/- and α7β2-/- mice was not inhibited by PNU282987 and MLA, confirming the absence of α7-containing nAChRs. DhβE and conotoxin MII were less effective against α7β2-/- and α3+/- mitochondria, due to the absence/decrease of β2- and α3-containing nAChRs, respectively (**Figure 9A**).

To test whether α9-containing nAChRs, up-regulated in the absence of α7 nAChR subunits, are involved in regulating cyt *c* release from mitochondria, we compared the effect of α-conotoxin PeIA in mitochondria of the WT and α7-/- mice. As shown in **Figures 9B,C**, α-conotoxin PeIA inhibited cyt *c* release from mitochondria stimulated by 0.9 μM Ca^2+^ and, to a lesser extent, by 0.5 mM H_2_O_2_ and its effect against Ca^2+^ was much more efficient in mitochondria of α7-/- than of the WT mice. Therefore, α9-containing nAChRs up-regulated in the absence of α7-containing ones could functionally substitute them to support mitochondria resistance to Ca^2+^.

## Discussion

The data presented here demonstrate the significance of mitochondrial nAChRs for supporting liver cells viability in the course of PHE or in nAChR subunit-deficient mice.

Partial hepatectomy, when 70% of the organ is being resected, is a classical and well-characterized model of mammalian tissue regeneration. Extensive damage of liver parenchyma stimulates hepatic cells to transit from quiescence to proliferation with eventual restoration of liver mass and function (Michalopoulos, 2010; Fausto et al., 2012). The consequence of events following PHE is well established. The first, priming phase of regeneration occurs during 3 h after surgery, while the DNA synthesis starts after 11–12 h (Kountouras et al., 2001). The initial period of regeneration is accompanied by inhibition of apoptosis and improving the survival rate of hepatocytes, usually attributed to the effect of transcription factors NFkB and STAT and inhibition of caspases 3 and 8 (Tarlá et al., 2006). We observed the increase of nAChR protein in liver mitochondria 3 h after PHE accompanied by decreased release of cyt *c* in response to either Ca^2+^ or H_2_O_2_ compared to mitochondria of intact liver. Instead, α7 and β2 subunits were decreased in mitochondria-depleted fraction during the priming phase of liver regeneration, starting to increase only after 6 h. This means that, after hepatectomy, the available nAChRs are targeted mainly to mitochondria and then the intracellular pool of nAChRs is replenished by *de novo* synthesis. By 12 h, the start of proliferative phase, the levels of nAChRs and cyt *c* released return to almost pre-operative state.

The properties of mitochondria during liver regeneration following PHE have been extensively studied. According to the literature data, in the early (pre-replicative) phase of liver regeneration, mitochondria ultrastructure is changed significantly: a number of mitochondria with dilated, paled and vacuolized matrix could be observed. The activities of Complex I+III, Complex II+III and Complex IV all decreased drastically at 6 h after hepatectomy and then gradually returned to the original level during 18–24 h (Tsai et al., 1992). The isolated mitochondria showed impairment in membrane permeability properties, an increase in Ca^2+^ content and the release of proteins, like aspartate aminotransferase and glutamic acid degydrogenase, from the matrix (Zhou et al., 2008). This suggests the occurrence in the early phase of liver regeneration of a transient mitochondrial oxidative stress accompanied with the changes in mitochondria ultrastructure. The mitochondrial ultrastructure, the membrane permeability properties and the Ca^2+^ content returned to normal values during the replicative phase of liver regeneration when a progressive recovery of liver mass is observed (Guerrieri et al., 2002). These data fit well with our observation showing the return of mitochondrial nAChR content to pre-operative state at 12 h after PHE. Despite alteration in mitochondrial membrane permeability properties, no release of cyt *c* was found during the prereplicative phase of liver regeneration (Guerrieri et al., 2002). This data indirectly confirm the stabilizing role of mitochondrial nAChRs during the critical period (3 and 6 h after PHE) when the remaining liver cells should be kept alive, as shown in our experiments.

Laparotomy does not damage the liver but includes stress reaction and local inflammation caused by the surgery (Markiewski et al., 2006). In contrast to PHE, all mitochondrial nAChR subunits, except α9, were decreased 3 and 6 h after LT. Consequently, liver mitochondria released more cyt *c* in response to either Ca^2+^ or H_2_O_2_ at 6 h time point.

These data clearly indicate that mitochondrial nAChRs are dramatically important during the initial phase of liver regeneration to support the survival of hepatic cells, but not their proliferation occurring later. In contrast, they are not involved in acute stress reaction when the liver is not damaged.

The importance of mitochondrial nAChRs was further confirmed in experiments with knockout mice, indicating that the absence or deficiency of α3-, α7-, or β2-containing nAChRs is compensated by α3β4, α4β4, α9α10, and α9β4 nAChR subtypes. Compared to α7β2 and α3β2 nAChRs, present in WT mitochondria, the compensating ones appeared to be less efficient against low but more efficient against high Ca^2+^ doses and, therefore, are expected to control the more severe apoptogenic challenges compared to those of the WT mice.

The data obtained in both knockout mice and operated rats allow discussing the impact of different nAChR subtypes in supporting mitochondria sustainability to apoptogenic stimuli. First of all, this concerns the α9- and β4-containing nAChRs, which are up-regulated in response to either nAChRs deficiency or tissue damage.

The α9 subunit belongs to the most ancient branch of nAChR genes evolutionary tree (Ortells and Lunt, 1995). Initially, the α9-containing nAChRs were found in the hair cells of the inner ear (Elgoyhen et al., 1994), but were further determined in many non-excitable cells (Chernyavsky et al., 2007; Colomer et al., 2010), the neurons of the dorsal root ganglia (Lips et al., 2002), the brain (Lykhmus et al., 2017), and mitochondria (Chernyavsky et al., 2007; Uspenska et al., 2017). The α9 subunits can contribute to both homomeric and heteromeric nAChRs (Plazas et al., 2005). In addition to previously described α9α10 combination, our data allow suggesting the presence of α9β4^∗^ nAChRs in the liver mitochondria of α7-/- and α7β2-/- mice. Taking into account that mitochondrial nAChRs do not function as ion channels (Gergalova et al., 2014), one can assume that mitochondria may contain the non-conventional nAChR subunit combinations, which do not form functional ion channels when expressed in the plasma membrane. However, this question, as well as the function and ligand specificity of α9β4 nAChRs, needs further examination. The up-regulation of α9 subunits was observed in keratinocytes (Chernyavsky et al., 2007), B-lymphocytes (Koval et al., 2011) and the brain of α7-/- mice (Lykhmus et al., 2017) that is in accord with our data.

A fast and dramatic increase of α9 nAChR subunits in the liver of both hepatectomized and laparotomized rats was, evidently, due to translation of available RNA messages. A large amount of α9 RNA was found in lymphocytes (Peng et al., 2004; Hao et al., 2011; Qian et al., 2011), although the α9 protein content was quite moderate (Koval et al., 2011). Therefore, it seems that this ancient alpha subunit is kept as a reserve and its amount can be quickly increased in response to environmental challenges, like stress or pain caused by the surgery. The α9-containing nAChRs were regarded as the targets to treat the chronic pain (Vincler and McIntosh, 2007; McIntosh et al., 2009); therefore, their up-regulation may be related to the pain reaction, which cannot be completely excluded in spite of an ether narcosis and analgesics used in our experiments.

The increase of α9-containing nAChRs in mitochondria of both operated rats and knockout mice resulted in their increased sensitivity to α-conotoxin PeIA. Interestingly, α-conotoxin PeIA affected mainly Ca^2+^-stimulated cyt *c* release in mitochondria of α7-/- mice, H_2_O_2_-stimulated cyt *c* release in mitochondria of hepatectomized rats and both in mitochondria of laparotomized rats. Previously we reported that Ca^2+^ or H_2_O_2_ induce cyt *c* release from mitochondria through different signaling pathways (Gergalova et al., 2014). The data presented here allow suggesting that α-conotoxin PeIA influenced non-identical pathways through α9α10 nAChRs (PHE), α9β4^∗^ nAChRs (α7-/- mice) or homomeric α9 nAChRs (LT). In general, the ability of α9^∗^ nAChRs to control various pathways demonstrates the universal nature of this ancient nAChR subtype that explains its urgent up-regulation in critical circumstances. The involvement of additional subunits seems to influence the signaling capacity of α9 homopentamer depending on the demand.

The up-regulation of β4-containing nAChRs was observed in mitochondria of α3+/-, α7-/-, and α7β2-/- mice. In these non-conditional knockouts, corresponding subunits are absent from the very beginning and, therefore, expression of other nAChR subunit genes can be regarded as a compensatory reaction.

The nature of beta subunits (β2 vs. β4) has been shown to influence the kinetic properties and agonist specificity of both α3-containing (Papke and Heinemann, 1991) and α4-containing nAChRs (Houlihan et al., 2001). Moreover, it was found that α3α5β4 nAChRs possess substantial Ca^2+^ permeability, comparable to that of α7 nAChRs (Lax et al., 2002). The β4-containing nAChRs are mainly expressed in autonomic ganglia neurons (Skok, 2002; Del Signore et al., 2004) and in certain brain areas (Grady et al., 2009), while, up to our knowledge, there is no information about their presence in non-excitable tissues. Here we show that the β4 subunit is expressed in the liver in the absence of α7, α3, or β2 nAChR subunits. However, since mitochondrial nAChRs were shown to function in an ion channel-independent manner (Gergalova et al., 2014), it is not clear whether the channel properties of β4-containing nAChRs may play a role.

Recently we reported that liver mitochondria of α7-/- mice become more sensitive to the non-competitive antagonist of α3β4^∗^ nAChRs (±)-18-methoxycoronaridine (18-MC) compared to mitochondria of the WT mice (Arias et al., 2015, 2018). Moreover, it was found that 18-MC, which affected mainly H_2_O_2_-stimulated cyt *c* release in mitochondria of the WT mice, became more efficient against Ca^2+^ in mitochondria of α7-/- mice. It was concluded that α3β4^∗^ nAChRs, up-regulated in the absence of α7-containing nAChRs, take their functional role to regulate Ca^2+^-stimulated apoptogenic events. Probably, α9β4^∗^ nAChRs, which appear in the absence of α7 subunits, are also intended to compensate the deficiency of CaKMII-dependent regulation in mitochondria. Previously, we observed a significant (several fold) up-regulation of β4 subunits in the brain mitochondria of α9-/- mice (Lykhmus et al., 2017). In contrast, as shown here, no significant changes were observed in β4-/- mitochondria, suggesting that in the WT mice its amount and function are negligible. Therefore, it seems that, along with the α9 subunit, the β4 one is a universal compensatory subunit, which starts to be expressed in mitochondria in the absence of α3, α7, α9, or β2 nAChR subunits and ensures the coupling of mitochondrial nAChRs to CaKMII-dependent signaling pathway.

## Conclusion

In conclusion, the two models used demonstrate the importance of mitochondrial nAChRs for supporting the viability of liver cells. The mitochondrial α3β2, α7β2, and α9α10 nAChRs are up-regulated during the priming phase of liver regeneration after PHE, and the absence or decrease of α7, β2, or α3 subunits in knockout mice are compensated by up-regulation of α3β4, α4β4, α7β4, α9β4, and α9α10 nAChRs to support the mitochondria resistance to apoptogenic influence. The α9 subunits are universal “reserve” ones, which are up-regulated both upon deficiency of other nAChR subtypes (in knockout animals) and in response to stress caused by tissue damage. Depending on the demand, they may be included in either homo- or hetero-meric receptors containing either α10 or β4 auxiliary subunits that underlies their involvement in intramitochondrial signaling. The β4 subunits start to be expressed in the absence of α7, β2, or α3 subunits to compensate the deficiency of mitochondrial CaKMII-dependent signaling. The concerted action of multiple nAChR subtypes controlling different signaling pathways in mitochondria ensures a reliable protection against apoptogenic factors of different nature.

Of course, the efficacy of anti-apoptotic protection depends not only on the quantity and subtypes of mitochondrial nAChR, but also on the availability of their natural ligands. The α7-containing nAChRs can be activated by choline, which is abundant in the cytosole and around mitochondria. In contrast, other nAChR subtypes require the presence of acetylcholine, which can be formed from choline by mitochondrial choline acetyltransferase (Pagliarini et al., 2008). Whether the quantity of intracellular acetylcholine or ChAT activity are altered in α7-/- mice and during PHE or LT is unknown and may be a subject of future studies. Consequently, mitochondrial nAChRs may be regarded as a promising pharmacological target for the therapy supporting the cell survival.

## Author Contributions

MS, MO, SK, and UM made substantial contributions to the conception or design of the work. KU, OL, MO, SP, and MS contributed to acquisition, analysis, and interpretation of data for the work. MS contributed to drafting the manuscript. KU, OL, MO, SK, and UM contributed to revising the manuscript critically for important intellectual content. KU, OL, MO, SP, UM, SK, and MS approved the final version to be published and agreed to be accountable for all aspects of the work in ensuring that questions related to the accuracy or integrity of any part of the work are appropriately investigated and resolved.

## Conflict of Interest Statement

The authors declare that the research was conducted in the absence of any commercial or financial relationships that could be construed as a potential conflict of interest.

## Abbreviations

CaMKII: calcium-calmodulin-dependent kinase
cyt *c*: cytochrome *c*
DhβE: dihydro-β-erythroidine
LT: laparotomy
MLA: methyllycaconitine
nAChR: nicotinic acetylcholine receptor
PHE: partial hepatectomy
